# Computerized Cognitive Training for Major Depressive Disorder: What’s Next?

**DOI:** 10.3389/fpsyt.2015.00137

**Published:** 2015-10-01

**Authors:** Jeffrey N. Motter, Davangere P. Devanand, P. Murali Doraiswamy, Joel R. Sneed

**Affiliations:** ^1^The Graduate Center, City University of New York, New York, NY, USA; ^2^Queens College, City University of New York, New York, NY, USA; ^3^Columbia University and the New York State Psychiatric Institute, New York, NY, USA; ^4^Duke University, Durham, NC, USA

**Keywords:** cognitive training, depression, geriatrics, executive dysfunction, processing speed

## Antidepressant Non-Response in Late-Life Depression

Major depressive disorder (MDD) affects 8–25% of adults aged 60 years and older and is a leading cause of morbidity and mortality in this population. Older individuals who are depressed have significantly increased risk of disability and death compared to older adults without depression. Treatment to remission is critical to mitigate the morbidity associated with MDD, as residual symptoms leave patients at increased risk of suicide, cardiovascular morbidity and mortality, and development of dementia. MDD occurring in the context of mild cognitive impairment (MCI; defined as 1.5 SDs below age and sex adjusted norms on memory) convert to dementia at a greater rate than elderly non-depressed patients with MCI (41 versus 15%) (1), lending support to the idea that late-life MDD may be a prodrome for later dementia (2). Although antidepressant medication is the most frequently used approach for treatment, as many as 50% of patients will not achieve remission with the first treatment (3). Furthermore, resolution of symptoms in late-life is often slower than in mid-life depression (4) and patients with a first depressive episode in late-life are also particularly likely to have a slow recovery compared to those with onset earlier in life (5). It is crucial, therefore, that we develop novel interventions to improve antidepressant response.

One of the most important factors in antidepressant non-response among older adults is the presence of executive dysfunction (ED) (5). ED refers to dysfunction in the executive function system, a broad class of mental functions necessary for adaptive and goal-directed behavior that includes planning, organization, problem solving, mental flexibility, and response inhibition (6). Impairment in frontostriatal-limbic networks, in particular the cognitive control network (CCN), is strongly implicated as mediating ED (7). The CCN, which consists of the anterior cingulate, dorsolateral prefrontal cortex, and their limbic connections, is involved in error detection and resolution. It has been implicated in impaired emotional processing (8). Distinct roles for the anterior cingulate cortex and dorsolateral prefrontal cortex have been delineated within the CCN: the anterior cingulate cortex monitors for the presence of response conflict processes and activates the dorsolateral prefrontal cortex to resolve the conflict using adjustment processes (i.e., such as the inhibition of responses to task-irrelevant stimuli) (9). Although the DLPFC and anterior cingulate cortex have distinct roles, they are interdependent components in the cognitive control process (10). Furthermore, damage to the ACC and DLPFC as well as impairment in the functional connectivity of the CCN is associated with poor antidepressant treatment response (11). However, successful antidepressant treatment appears to alleviate impairment in the CCN (8). Taken together, it appears that antidepressant treatment non-response is associated with ED and may be mediated (at least partially) by integrity of the CCN.

## Treatment of ED in Late-Life Depression

Two important treatment options have been developed that target ED in geriatric depression: problem-solving therapy (PST) (12) and computerized cognitive training (CCT) (13). PST consists of 12 weekly, 1-hour psychotherapy sessions aimed at improving problem-solving abilities that rely on executive functions. PST has been shown to be more effective than supportive psychotherapy among depressed older adults with ED (14). However, PST is limited in that it is time consuming, requires specialized training, and is therefore not readily available in most areas.

Using CCT to target executive functions and improve frontolimbic connectivity has also been suggested as a potential treatment approach for ED in geriatric depression and has received some attention in the literature (15). CCT consists of computerized cognitive exercises used to target neural networks to improve cognitive functioning through neuroplasticity. As it can be completed at home, CCT creates fewer disruptions in a patient’s life than on-site treatments. Compared to existing treatments, it is easily accessible, inexpensive, non-invasive, and scaled to the skill level of each individual. CCT, therefore, is consistent with the goal of personalizing medicine (16). There is also no concern for medicinal side effects or drug × drug interactions, and limitations due to participant mobility are significantly mitigated. Compliance with CCT is also easily monitored and documented CCT is also considerably less time consuming than augmentation with psychotherapy. CCT, in research studies, commonly takes 2–10 weeks, with sessions usually lasting between 30 and 60 min (17–23), although it is optimal dosing is not yet determined. PST, on the other hand, typically lasts 12 weeks and consists of hourly sessions (14). However, it should be noted that highly effective, short, self-administered, web based cognitive and problem-solving psychotherapy modules are also available. In all, CCT is a promising augmentation strategy for antidepressant medication (13, 15).

Computerized cognitive training has demonstrably produced functional changes in neural networks in a variety of patient populations (24) that are linked with both improved cognitive performance during tasks (25) and improved regional local efficiency at rest (26). Targeted training of executive functions in older adults has been associated with increased resting cerebral blood flow in the prefrontal cortex (27). When targeting the CCN, CCT focused on increasing executive functioning has resulted in improved regional local efficiency in the left middle frontal gyrus as well as higher degree and shorter path length in the left superior frontal gyrus. These changes in interactivity and integration are indicative of reorganization of functional connections (26).

Of the studies that have been conducted using CCT for depression, many have found consistent improvements in working memory, attention, and mood (17, 18, 20, 23, 28, 29). However, increases in executive functioning are found in some studies (30), but not in all outcome measures of others (20). Even though ED is a critical component of late-life depression, it is rarely a cornerstone of CCT paradigms. Perhaps more notably absent as both a training target and outcome measure is processing speed (PS).

The omission of PS is important because executive processes are by definition complex, higher order mental operations that depend on the integration of more simple, lower-order cognitive processes (31). One possibility, therefore, is that a lower-order cognitive process, such as PS, may account for the effects of ED on antidepressant treatment response (32). For example, a decrease in PS may prevent executive processes from being successfully executed when relevant operations cannot be successfully executed within the necessary time frame or when the products of early processing are not available for later processing (33). Indeed, several studies have shown that PS mediates performance on tests of executive functioning in geriatric depressed patients (34, 35). One study in particular showed that neuropsychological deficits in all cognitive domains were mediated by slowed PS in a sample of depressed older adults (36).

## Model of Improved Antidepressant Response

**Figure 1:**
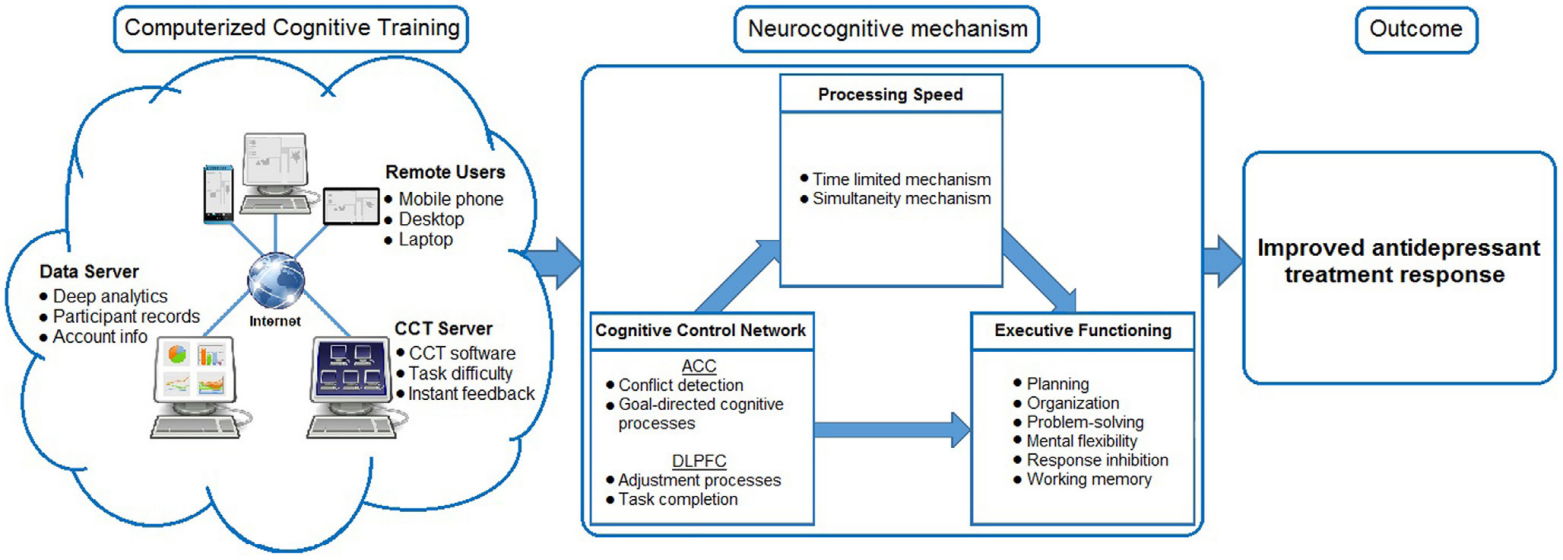
**Model of improved antidepressant response**.

Our proposed method for improving antidepressant non-response in older adults is presented in Figure 1. At the core of the model is PS, which some have argued is the “the core cognitive deficit in LLD” and that deficits in PS underlie ED (37). If information is processed too slowly, executive functions cannot be performed. This is known as the limited time mechanism (33). Furthermore, products of earlier processes may be no longer available when later processes are complete. As executive functions require the integration of multiple sources of information, impaired PS disallows these inputs from being incorporated into decision-making. This is known as the simultaneity mechanism (33).

Processing speed deficits lead to multiple downstream consequences for executive functioning, mood, and everyday functioning. Decreased PS has been repeatedly found in older patients with depression relative to healthy controls and mediates the effects of depression and ED (38). Decreased PS places individuals on a trajectory of poor health outcomes, including an increased risk of dementia, the development of functional dependence in activities of daily living (ADL), and driving cessation. We recently showed that PS is central to the effect that depression and ED have on functional impairment in cognitively impaired older adults (39). Using data from the National Alzheimer’s Coordinating Center, we identified PS as central to the effect that depression and ED have on functional impairment in cognitively impaired older adults. Slowing as measured by Digit Symbol Substitution Test partially mediated the effect of depression on daily functioning (5.8% of the direct effect of depression on function was explained by Digit Symbol) and fully mediated the effect of ED on function (91.7% of the direct effect of Trailmaking Test B after controlling for the effect of Trailmaking Test A was explained by slowing on Digit Symbol). Indeed, the interrelatedness of PS, mood, and everyday functioning is further reinforced by the finding that CCT focused on PS produces increased odds of reduced depressive symptoms as well as reduced odds of developing impairments in factors of daily living (40). Taken together, we propose that CCT targeting PS may lead to improved functioning of the CCN, amelioration of ED, and in turn, improved treatment response.

## Methodological Issues in CCT

Targeting PS with novel interventions that aim to improve functional connectivity in the CCN has the potential to significantly change the future treatment of depression. It is important to note that while CCT may alleviate cognitive deficits, it is not a remedy for factors precipitating the onset of depression, such as social isolation, bereavement, inflammation, and underlying medical conditions. The implementation of CCT for late-life depression is met with several challenges. Only 58.3% of older adults have home internet access (41), making CCT difficult to implement in all cases. Researchers and clinicians must be prepared to make accommodations for those unable to participate in CCT at home. Increased availability of CCT via phones might help in this regard. Previous studies have relied on either waitlist control conditions or control conditions that do not account for engagement and motivation. This is a major methodological limitation because loss of interest and motivation are hallmarks of depression. Not only might these placebo-like factors interfere with the implementation of CCT but also should positive effects on mood be found, it is difficult to know whether they are specific to CT or are results of participation in a rewarding activity. Rigorously controlled studies with comparison groups are needed to clarify whether the effects obtained are specific to CCT.

Another problem plaguing the success of previous CCT programs is the failure to transfer cognitive gains as a result of CCT to other laboratory tasks or day-to-day functioning. Transfer to everyday functioning is particularly important given the strong association between depression and disability. Most studies do not assess transfer of cognitive improvement to everyday function and quality of life, and therefore, can be criticized for teaching to the test (i.e., training patients in games that are similar to the outcome measure). Although there is some evidence that CCT gains in one cognitive domain may transfer to other, which closely related cognitive domains (42), studies demonstrating that CCT gains transfer to everyday functioning are few and far between. Assessing transfer to everyday functioning is a critical yet often neglected component of CCT studies, as the merits of increased cognitive functioning in the absence of improved everyday functioning or mood are questionable. Though there is promising evidence that suggests CCT based in PS creates far transfer to both everyday functioning and mood for healthy older adults (40), this has yet to be demonstrated for depressed older adults.

## Looking Forward

Exploring the viability of PS training for late-life depression will help answer broader questions about the efficacy of CCT. In addition to elderly patients with depression, younger adults with depression may also benefit from CCT as has been shown in initial studies (21, 28). CCT needs to be compared with psychotherapy and to newer techniques, such as problem-solving therapy and possibly neurofeedback training. The popularity of CCT among consumers and the claims of manufacturers has led to opposing groups of scientists criticizing (43) and defending (44) the value of CCT, emphasizing the importance of well-designed randomized controlled trials to objectively evaluate helpfulness in a high-risk group. This line of study will also consider the feasibility of targeting specific neural networks to restore functioning. Understanding the relationship between improved cognition and network integrity begins to bridge the gap between neuroimaging and clinical practice.

## Conflict of Interest Statement

Davangere P. Devanand has received fees for scientific advisory boards from AbbVie, Lundbeck, and Intracellular Therapeutics. P. Murali Doraiswamy has received advisory/speaking fees and research grants from several health and biotechnology companies. He owns stock in Anthrotronix, Muses Labs, and Adverse Events, Inc. whose products are not discussed here. The other co-authors declare that the research was con­ducted in the absence of any commercial or financial relationships that could be construed as a potential conflict of interest.
